# Does the Interaction Between Alcohol Use and Depression Exacerbate Hyperglycemia Risk? A Cross-Sectional Study Beyond Additive Effects

**DOI:** 10.3390/medicina61081380

**Published:** 2025-07-30

**Authors:** Simona-Dana Mitincu-Caramfil, Anca Pantea Stoian, Lavinia-Alexandra Moroianu, Catalin Plesea-Condratovici, Andrei Vlad Bradeanu, Eduard Drima

**Affiliations:** 1 Pharmaceutical Sciences Department, Faculty of Medicine and Pharmacy, “Dunărea de Jos” University, 800201 Galaţi, Romania; simona_caramfil@yahoo.com; 2 Doctoral School of Biomedical Sciences, “Dunărea de Jos” University, 800201 Galați, Romania; 3 Department of Diabetes, Nutrition and Metabolic Diseases, Carol Davila University of Medicine and Pharmacy, 020021 Bucharest, Romania; ancastoian@yahoo.com; 4 Morphological and Functional Sciences Department, Faculty of Medicine and Pharmacy, “Dunărea de Jos” University, 800201 Galaţi, Romania; 5 Surgical Department, Faculty of Medicine and Pharmacy, “Dunărea de Jos” University, 800201 Galaţi, Romania; andrei.bradeanu@ugal.ro; 6 Clinical Medical Department, Faculty of Medicine and Pharmacy, “Dunărea de Jos” University, 800201 Galaţi, Romania; drima_edi1963@yahoo.com

**Keywords:** alcohol, depression, hyperglycemia, diabetes mellitus

## Abstract

*Background and Objectives*: This study investigated whether the interaction between heavy alcohol use and depression amplifies the risk of hyperglycemia in psychiatric patients. *Materials and Methods*: We conducted a cross-sectional study on 172 patients (aged 18–65) hospitalized at the “Elisabeta Doamna” Clinical Psychiatric Hospital, Romania. The data included fasting blood glucose, gamma-glutamyl transferase (GGT), Beck Depression Inventory (BDI), and Alcohol Use Disorders Identification Test (AUDIT) scores. *Results*: Moderate positive correlations were observed between depression scores and blood glucose (r = 0.44) and between alcohol consumption and blood glucose (r = 0.43). The interaction term (BDI × AUDIT) was statistically significant in multiple regression (β = 0.012, *p* = 0.001), and the model explained 39.1% of glucose variability. Logistic regression analysis revealed that neither high alcohol consumption (OR = 1.38, *p* = 0.441) nor severe depression alone (OR = 1.30, *p* = 0.582) were significantly associated with hyperglycemia. However, their interaction demonstrated a strong and statistically significant effect (OR = 19.3, 95% CI: 3.22–115.81, *p* = 0.001). The prevalence of hyperglycemia reached 95.8% in patients with both risk factors. *Conclusions*: The combined presence of high alcohol consumption and severe depression significantly increases the risk of hyperglycemia. These findings highlight the importance of integrated screening and interventions in psychiatric settings.

## 1. Introduction

Type 2 diabetes mellitus (T2DM) represents a major public health concern worldwide, with a steadily increasing prevalence [1,2]. In Romania, recent estimates suggest a national diabetes prevalence of 11.6% among the adult population [3,4]. Classic risk factors include obesity, physical inactivity, family history, and unbalanced diet; however, recent research has increasingly recognized the contribution of psychological and behavioral factors in the pathogenesis of this condition [5,6].

Depression and heavy alcohol use are frequent in psychiatric practice and have each been associated with an increased risk of developing T2DM [6,7]. These associations may involve both behavioral mechanisms (e.g., poor dietary choices, reduced physical activity, low treatment adherence) and physiological pathways such as hypothalamic–pituitary–adrenal (HPA) axis dysregulation, systemic inflammation, and insulin resistance [7,8].

Several studies have reported that depression and excessive alcohol consumption can individually impair glucose metabolism by reducing insulin sensitivity and disrupting insulin secretion [9,10,11].

A less explored area concerns the combined effect of these two factors. Although depression and alcohol misuse frequently co-occur and reinforce each other, few studies have examined their interaction effect on metabolic outcomes such as hyperglycemia or diabetes [12,13,14,15,16,17,18].

Considering this gap in the literature, the present study aimed to investigate the relationship between alcohol consumption, depression, and blood glucose levels in a psychiatric inpatient sample from the “Elisabeta Doamna” Clinical Psychiatric Hospital in Galați, Romania. Specifically, we evaluated whether the combination of heavy alcohol use and severe depression amplifies the risk of hyperglycemia beyond their contributions. To our knowledge, this is one of the first studies to statistically quantify this interaction effect in such a clinical context.

Although the independent associations between depression, alcohol consumption, and impaired glucose metabolism have been documented, little is known about their potential synergistic interaction. Most existing studies analyze these factors in isolation. To our knowledge, this is one of the first studies to evaluate the statistical interaction between severe depression and heavy alcohol use as combined risk factors for hyperglycemia in a psychiatric inpatient population. By quantifying this interaction effect, our study offers novel insights that may inform screening and intervention strategies in vulnerable clinical populations.

Although previous research has independently linked depression and excessive alcohol consumption to alterations in glucose metabolism, few studies have examined their potential interaction. The possibility that these two factors may act synergistically to amplify the risk of hyperglycemia remains underexplored. This study aims to fill this gap by investigating whether the co-occurrence of high alcohol intake and severe depression is associated with a significantly greater risk of hyperglycemia in a psychiatric inpatient population. To our knowledge, this is one of the first studies to quantify the interaction effect between these two risk factors in this specific clinical context.

## 2. Materials and Methods

### 2.1. Study Design and Participants

A cross-sectional observational study was conducted between 2021 and 2023 at the “Elisabeta Doamna” Clinical Psychiatric Hospital in Galaţi, Romania. The final sample consisted of 172 hospitalized patients (113 men and 59 women) aged between 18 and 65 years.

Inclusion criteria were age over 18 years, the ability to provide informed consent, and no prior diagnosis of diabetes mellitus.

Exclusion criteria included pregnancy, active malignancy, severe hepatic disorders, cognitive impairments affecting questionnaire reliability, and a history of alcohol dependence or addiction as documented in the patients’ medical records. Additionally, individuals with a known diagnosis of diabetes or receiving glucose-lowering treatment were excluded.

These criteria were applied to minimize potential confounders that could independently affect glucose metabolism, such as underlying metabolic disorders or severe somatic illness. Cognitive impairment was also excluded to preserve the validity of self-reported data (BDI and AUDIT data).

Out of the 212 patients initially screened, 40 were excluded based on these criteria, resulting in a final analytic sample of 172 participants.

The study was approved by the Ethics Committee of the “Elisabeta Doamna” Clinical Psychiatric Hospital (approval code P.O. 2.2-STUD 01, decision no. 4, 22 October 2019) and was conducted in accordance with the Declaration of Helsinki and good clinical practice guidelines. This paper is part of the doctoral research of psychologist Simona-Dana Mitincu-Caramfil (The role of ethanol consumption in the development of depressive syndrome). Data extraction forms and processed data are available from the corresponding author upon reasonable request. No unpublished materials were used.

### 2.2. Data Collection and Measurements

Demographic and clinical data were collected using a standardized questionnaire, which included information on age, sex, education, marital status, personal medical history, and current medication. Anthropometric measurements included height, weight, and waist circumference, and the body mass index (BMI) was calculated using the following standard formula: weight (kg)/height^2^ (m^2^).

Fasting venous blood samples were collected via standard venipuncture within the first 24 h after admission, between 7:00 and 9:00 AM, following a minimum of 8 h of overnight fasting. Plasma glucose levels were determined using the glucose oxidase method. Gamma-glutamyl transferase (GGT) was also measured from the same fasting sample.

Values ≥ 140 mg/dL were considered indicative of hyperglycemia, based on World Health Organization epidemiological criteria for intermediate hyperglycemia and diabetes risk stratification. All participants were not receiving any glucose-lowering treatment, and individuals with a known diagnosis of diabetes mellitus were excluded [19].

### 2.3. Assessment of Depression and Alcohol Consumption

Depression was assessed using the BDI, a validated instrument consisting of 21 items that measure the severity of depressive symptoms. BDI scores were interpreted as follows: 0–10 = minimal depression, 11–20 = mild depression, 21–30 = moderate depression, and 31–40 = severe depression. For categorical analyses, a BDI score greater than 25 was considered indicative of severe depression [20,21,22].

Alcohol consumption was evaluated using the AUDIT, an instrument developed by the World Health Organization. AUDIT scores were interpreted as follows: <7 = low-risk consumption, 8–14 = hazardous consumption, 15–20 = harmful consumption, and ≥20 = alcohol dependence. For categorical analyses, an AUDIT score greater than 20 was considered indicative of high-risk alcohol consumption, consistent with the WHO classification for alcohol dependence [23,24].

### 2.4. Statistical Analysis

The data were analyzed using Python and associated statistical packages. Descriptive statistics were calculated for all variables of interest. The normality of distributions was assessed using the Kolmogorov–Smirnov test. All key continuous variables followed an approximately normal distribution, which supported the use of parametric statistical tests. Group comparisons were conducted using Student’s *t*-test for continuous variables and the chi-square test for categorical variables.

A one-way analysis of variance (ANOVA) was used to compare mean blood glucose levels across groups defined by combinations of alcohol consumption and depression severity. When the overall ANOVA was statistically significant (*p* < 0.05), post hoc pairwise comparisons were conducted using Tukey’s Honest Significant Difference (HSD) test, with adjustments for multiple comparisons.

All statistical analyses and visualizations were performed using Python version 3.10.11 in the Thonny 4.1.4 environment, running on Windows 11. Data processing was conducted using the pandas library (version 1.5.3), and numerical operations were performed with numpy (version 1.23.5). Statistical tests, including correlation analyses and ANOVA, were performed using scipy (version 1.10.1) and statsmodels (version 0.13.5). Visualizations, including the correlation matrix and boxplots, were created using matplotlib (version 3.7.1) and seaborn (version 0.12.2). Logistic regression models and classification metrics were implemented using scikit-learn (version 1.2.2).

Correlations between continuous variables were assessed using Pearson’s correlation coefficient. Multiple linear regression was used to evaluate the relationship between the independent variables (alcohol consumption, depression score, and GGT) and fasting blood glucose level. To estimate the risk of hyperglycemia, logistic regression models were used to calculate odds ratios (ORs) and 95% confidence intervals (CIs).

To evaluate the interaction between alcohol use and depression, an interaction term was included in the regression models, and stratified subgroup analyses were performed. A two-tailed *p*-value < 0.05 was considered statistically significant for all analyses.

All logistic regression models were adjusted for age, sex, and gamma-glutamyl transferase (GGT) levels to account for potential confounding.

## 3. Results

### 3.1. Demographic and Clinical Characteristics

The final sample included 172 participants, of whom 113 (65.7%) were men and 59 (34.3%) were women, with a mean age of 43.5 ± 12.3 years.

The prevalence of heavy alcohol consumption (AUDIT score >20) in the study sample was 54.1%, being more frequent in men (59.3%) compared to women (45.8%), although the difference did not reach statistical significance (*p* = 0.126). Severe depression (BDI score >25) was present in 45.9% of participants, with a slightly higher prevalence in women (52.5%) compared to men (39.8%), but again, the difference was not statistically significant (*p* = 0.152).

Hyperglycemia (blood glucose > 140 mg/dL) was identified in 58.7% of participants, being more frequent in men (61.9%) compared to women (52.5%), although the difference was not statistically significant (*p* = 0.305). The mean blood glucose level in the entire sample was 144.7 ± 26.2 mg/dL, and the mean GGT level was 59.7 ± 17.6 U/L.

Descriptive statistics for the study population’s baseline characteristics are presented in Table 1.

### 3.2. Correlations Between Variables of Interest

**Figure 1:**
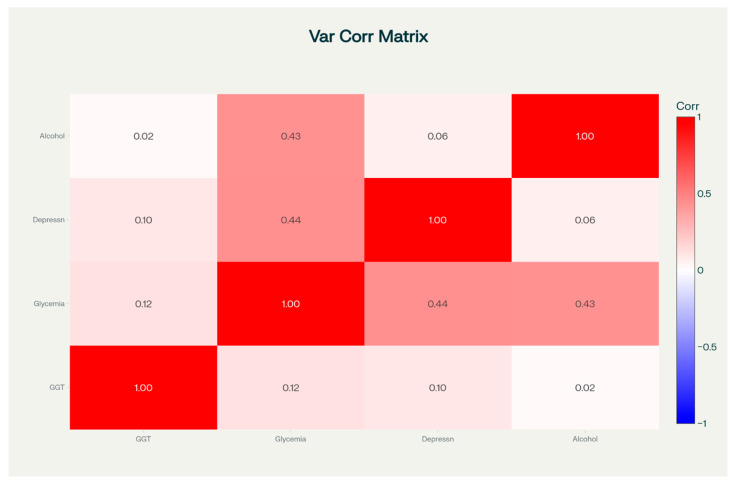
The Pearson correlation matrix among fasting blood glucose, gamma-glutamyl transferase (GGT), the Beck Depression Inventory (BDI) score, and the Alcohol Use Disorders Identification Test (AUDIT) score in the study population. The matrix displays Pearson correlation coefficients, with the strength of associations visually represented by the intensity of the red shading—darker tones indicate stronger correlations. The diagonal shows perfect correlations (r = 1.00) for each variable with itself. Notably, moderate positive correlations were observed between blood glucose and the depression score (r = 0.44) and between blood glucose and the alcohol consumption score (r = 0.43). In contrast, correlations involving GGT were weak and not statistically significant.

The Pearson correlation matrix among fasting blood glucose, gamma-glutamyl transferase (GGT), depression scores (the BDI), and alcohol consumption (the AUDIT) is presented in Figure 1.

**Figure 2:**
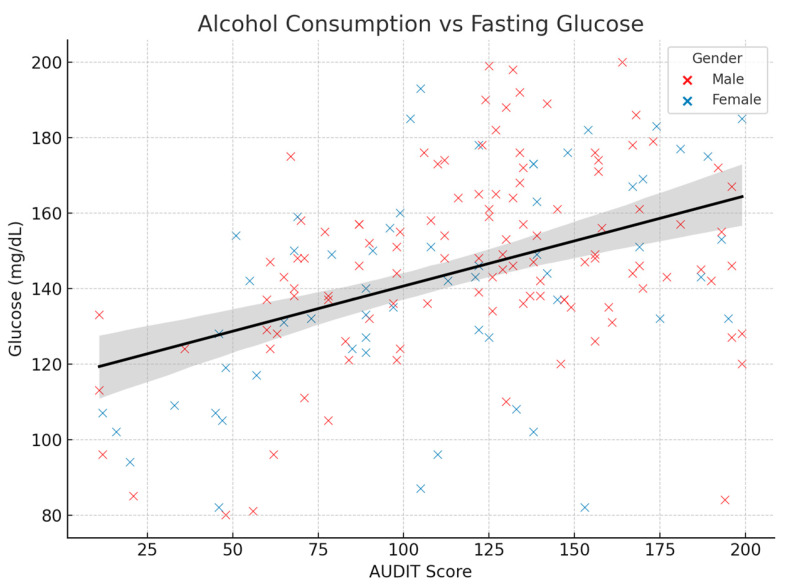
Relationship between alcohol consumption (measured by audit score) and blood glucose level in study patients.

Figure 2 displays the scatter plot illustrating the relationship between alcohol consumption (measured by the AUDIT score) and blood glucose levels in the 172 study participants from the *“Elisabeta Doamna”* Clinical Psychiatric Hospital. Each point represents an individual patient, with blue dots indicating male patients and orange dots representing female patients. The gray trend line demonstrates a positive correlation (r = 0.43, *p* < 0.001) between alcohol consumption and blood glucose levels. Higher AUDIT scores are associated with elevated blood glucose levels across both genders, supporting the study’s findings of a significant association between alcohol consumption and hyperglycemia risk. This figure effectively visualizes the moderate positive correlation between alcohol consumption and blood glucose levels that was identified in the statistical analysis, showing how patients with higher alcohol consumption scores tend to have higher blood glucose levels regardless of gender.

Interestingly, no significant correlation was observed between GGT levels and the AUDIT score (r = 0.02, *p* = 0.754), contrary to expectations based on the literature, which suggests that GGT is a sensitive marker for chronic alcohol consumption. This lack of association may be explained by factors such as pre-existing liver conditions or individual variability in alcohol metabolism.

**Figure 3:**
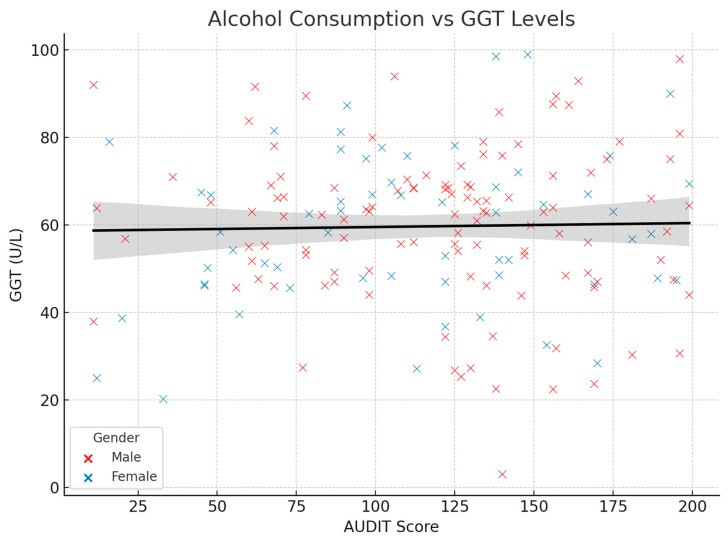
Scatter plot showing relationship between alcohol consumption (AUDIT score) and GGT enzyme levels in study patients, color-coded by gender, with regression trend line.

Figure 3 displays the scatter plot illustrating the relationship between alcohol consumption (measured by the AUDIT score) and GGT enzyme levels in the 172 study participants from the “Elisabeta Doamna” Clinical Psychiatric Hospital. Each point represents an individual patient, with blue dots indicating male patients and orange dots representing female patients. The gray regression line shows a weak, nonsignificant correlation (r = 0.02, *p* = 0.754) between alcohol consumption scores and GGT levels. Contrary to expectations from the literature suggesting GGT as a sensitive marker of chronic alcohol consumption, this study found no significant association between AUDIT scores and GGT enzyme levels, which may be explained by pre-existing liver conditions or individual variability in alcohol metabolism among the psychiatric patient population.

To further explore the relationships between key variables, we performed a stratified correlation analysis by sex. In both men and women, alcohol consumption was positively associated with fasting blood glucose, with a slightly stronger correlation observed in women (r = 0.51, *p* < 0.001) compared to men (r = 0.36, *p* = 0.0001). Similarly, the association between depression scores and glycemia remained statistically significant in both groups, with a correlation of *r* = 0.48 (*p* < 0.001) in men and *r* = 0.44 (*p* = 0.0005) in women.

The correlation between alcohol and depression was weak and not statistically significant in either group (*r* = 0.14, *p* = 0.13 for men; *r* = −0.04, *p* = 0.78 for women). Interestingly, the association between alcohol use and GGT levels was weak and nonsignificant in both sexes (*r* = −0.06, *p* = 0.50 in men; *r* = 0.17, *p* = 0.19 in women). These results suggest that the observed positive associations between alcohol use, depression, and glycemic status are consistent across sexes, but slightly stronger in women.

### 3.3. Glycemia Levels by Depression Category and Alcohol Consumption

**Figure 4:**
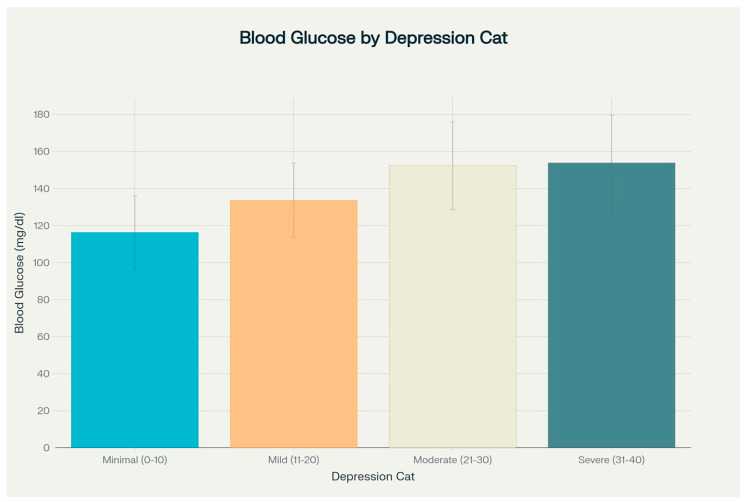
Distribution of blood glucose levels by depression category.

The analysis of glycemia levels according to the severity of depression revealed a clear trend of increasing mean glycemia values with higher levels of depressive symptoms. Patients with minimal depression (BDI score 0–10) had a mean glycemia of 116.3 ± 19.8 mg/dL; those with mild depression (BDI score 11–20) had 133.6 ± 20.1 mg/dL; patients with moderate depression (BDI score 21–30) had 152.4 ± 23.6 mg/dL; and those with severe depression (BDI score 31–40) had 153.9 ± 25.6 mg/dL. The one-way ANOVA confirmed that these differences were statistically significant (F = 17.81, *p* < 0.001). The distribution of glycemia levels according to depression category is illustrated in Figure 4

**Figure 5:**
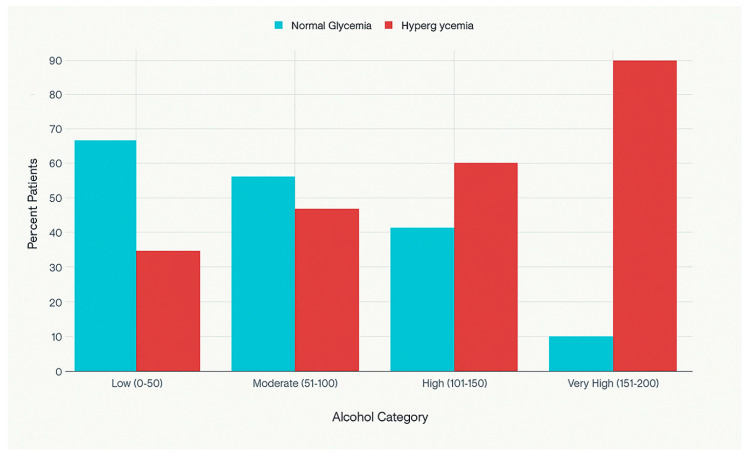
Prevalence of hyperglycemia by alcohol consumption level.

Similarly, a relationship was observed between the categories of alcohol consumption and blood glucose levels. Patients with low alcohol consumption (0–50 g/day) exhibited an average blood glucose level of 105.6 ± 16.3 mg/dL, those with moderate consumption (51–100 g/day) had 137.6 ± 17.7 mg/dL, those with high consumption (101–150 g/day) had 153.8 ± 24.6 mg/dL, and those with very high consumption (151–200 g/day) had 152.7 ± 25.0 mg/dL. Variance analysis confirmed the statistical significance of these differences (F = 22.44, *p* < 0.001). The prevalence of hyperglycemia based on the level of alcohol consumption among the study patients is presented in Figure 5.

**Figure 6:**
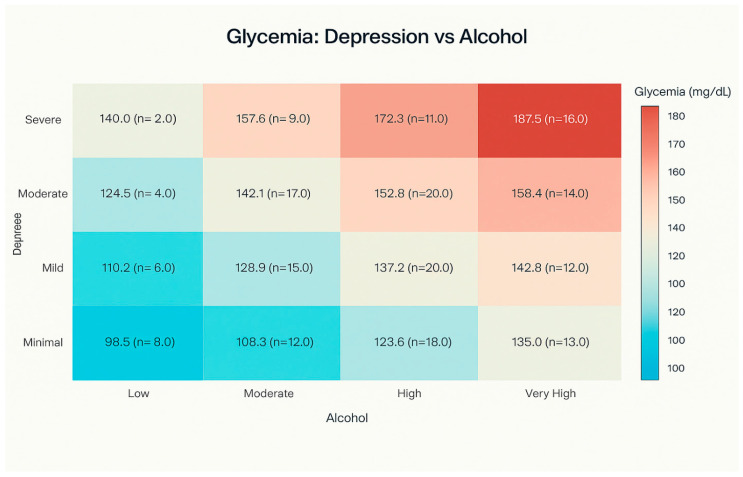
Mean blood glucose level by depression severity and alcohol consumption category.

The two-dimensional analysis of mean blood glucose levels for different combinations of depression and alcohol consumption categories revealed a significant interaction effect. The highest mean blood glucose level (169.0 ± 3.2 mg/dL) was observed in patients with severe depression and extremely high alcohol consumption, while the lowest mean level (95.8 ± 6.2 mg/dL) was recorded in patients with severe depression but low alcohol consumption. The mean blood glucose level by depression severity and alcohol consumption in study patients is presented in Figure 6.

### 3.4. Prevalence of Hyperglycemia by Risk Factors

An analysis of the prevalence of hyperglycemia (glycemia > 140 mg/dL) by risk factor presence revealed significant differences between groups. In the group without risk factors (no severe depression and no heavy alcohol consumption), the prevalence of hyperglycemia was 40.0%. In the group with severe depression but no heavy alcohol consumption, the prevalence increased slightly to 46.4%, and in the group with heavy alcohol consumption but no severe depression, the prevalence was 47.8%.

Notably, in the group with both risk factors (both severe depression and heavy alcohol consumption), the prevalence of hyperglycemia was significantly higher, reaching 95.8%. This dramatic increase suggests a strong interaction effect between the two risk factors.

**Figure 7:**
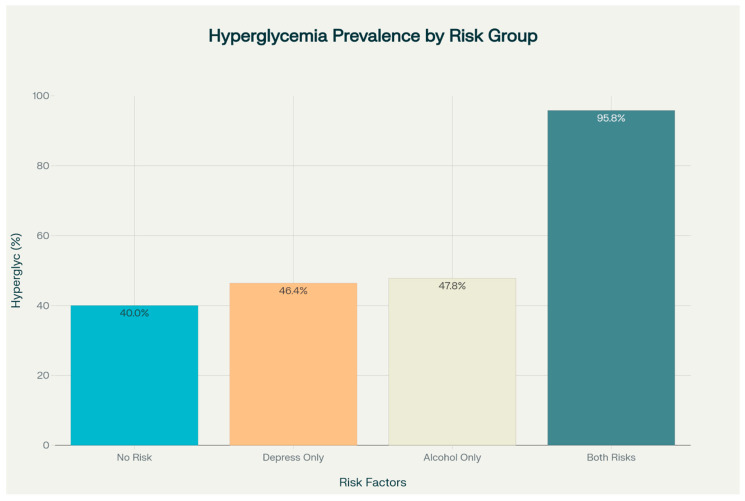
Prevalence of hyperglycemia by presence of high alcohol consumption and depression in studied patients.

Figure 7 displays the prevalence of hyperglycemia across four distinct patient groups categorized by the presence or absence of high alcohol consumption and severe depression. The chart demonstrates a dramatic synergistic effect, with patients having both risk factors showing a prevalence of 95.8%, significantly higher than those with only one risk factor (46.4% for depression alone and 47.8% for high alcohol consumption alone) or no risk factors (40.0%).

### 3.5. Regression Analysis and Risk Estimation

Multiple linear regression analysis confirmed the independent association of both depression score and alcohol consumption with blood glucose level. In the model adjusted for age, sex, and GGT, both the BDI score (β = 1.19, *p* < 0.001) and the AUDIT score (β = 0.22, *p* < 0.001) remained significant predictors of blood glucose. The full model explained 37.5% of the variance in blood glucose levels (R^2^ = 0.375, *p* < 0.001).

A particularly interesting finding emerged when the interaction term (BDI × AUDIT) was added to the multiple linear regression model, which proved to be statistically significant (β = 0.012, *p* = 0.001), indicating that the effect of depression on blood glucose is moderated by alcohol consumption and vice versa. The interaction model explained 39.1% of the variance in blood glucose (R^2^ = 0.391, *p* < 0.001).

In the logistic regression analysis adjusted for age, sex, and GGT levels, neither high alcohol consumption (OR = 1.38, *p* = 0.441) nor severe depression (OR = 1.30, *p* = 0.582) were significantly associated with hyperglycemia. However, their interaction term showed a strong and statistically significant association (OR = 19.30, 95% CI: 3.22–115.81, *p* = 0.001). Table 2 summarizes these results.

A complete overview of all logistic regression models, including covariates and confidence intervals, is presented in Table 2.

In addition, a separate logistic regression analysis comparing patients with both risk factors (high alcohol use and severe depression) to those with neither revealed an adjusted odds ratio of 28.85 (95% CI: 4.81–173.02, *p* < 0.001), indicating a substantial cumulative effect on hyperglycemia risk.

### 3.6. Relationship Between Risk Factors and Mean Glycemia Levels

Table 3 presents mean blood glucose levels stratified by the presence or absence of high alcohol consumption and severe depression. Although the table presents descriptive group means, statistical analysis using ANOVA confirmed that the differences across groups were statistically significant (*p* < 0.001), supporting the interaction effect described in Section 3.5.

Patients with no risk factors had a mean glycemia level of 133.9 ± 25.9 mg/dL, those with only severe depression had a mean of 134.5 ± 24.1 mg/dL, and those with only high alcohol consumption had a mean of 143.3 ± 25.5 mg/dL. In contrast, patients presenting both risk factors showed a significantly higher mean glycemia level of 163.3 ± 17.4 mg/dL (*p* < 0.001 compared to the other groups). The relationship between risk factors and mean glycemia levels is detailed in Table 3, while the mean glycemia values according to alcohol consumption and depression severity are illustrated in Figure 7.

### 3.7. Mediation Analysis

To explore whether the effect of alcohol consumption on fasting blood glucose is mediated by depression severity, a mediation analysis was performed using a three-step approach. First, the relationship between alcohol consumption (the AUDIT score) and depression severity (the BDI score) was assessed, showing a positive and statistically significant association (path a: β = 0.012). Second, depression severity was found to be positively associated with fasting glucose levels while controlling for alcohol consumption (path b: β = 1.19). Third, the total effect of alcohol on blood glucose (path c) was β = 0.24, whereas the direct effect, controlling for depression (path c′), was β = 0.23.

The indirect effect (a × b) of alcohol on glucose via depression was 0.0142. However, this indirect pathway was not statistically significant as the 95% confidence interval obtained via bootstrap resampling (1000 iterations) ranged from −0.0254 to 0.0487, thus including 0. These findings suggest that, while alcohol consumption is associated with higher depression scores and depression is related to elevated glucose levels, depression does not significantly mediate the relationship between alcohol use and hyperglycemia in this psychiatric inpatient sample.

## 4. Discussion

Our results, which highlight a strong interaction between alcohol consumption, depression, and the risk of hyperglycemia during 2021–2023, gain additional depth when placed in the recent context of the COVID-19 pandemic. A previous study conducted in the same hospital unit compared the years 2019 and 2020, observing the direct impact of the pandemic on anxiety and depressive disorders [25]. Interestingly, the authors reported a decrease in cases associated with ethanol consumption in 2020 compared to 2019, suggesting that factors such as quarantine and increased family integration may have played a temporarily protective role. In this context, the high prevalence and devastating synergistic effect observed in our study (2021–2023) may reflect not only a return to pre-pandemic levels but an exacerbation of these risk behaviors as a coping mechanism in the post-pandemic period [26,27,28]

Moreover, the vulnerability observed in the studied adult population could represent the culmination of risk trajectories initiated early in life. The literature, including recent meta-analyses, emphasizes that childhood obesity—a major precursor of adult diabetes—is deeply interconnected with psychological factors and was exacerbated by the pandemic context [29]. This suggests that our patients, who present with alcohol–depression comorbidity, may have a history of metabolic and psychological vulnerability dating back to childhood. Thus, the devastating synergistic effect observed in adulthood is not an isolated phenomenon but the result of a long-term accumulation of biological, psychological, and behavioral risk factors.

The distinction between the interaction model and the two-vs-zero risk factors model strengthens the robustness of our findings, both adjusted for key covariates (age, sex, and GGT).

### 4.1. The Association Between Alcohol Consumption, Depression, and Hyperglycemia in the Context of the Scientific Literature

Our study demonstrates a significant interaction between alcohol consumption and depression in predicting hyperglycemia, aligning with a substantial body of the scientific literature that explores these complex relationships, including pilot studies conducted in young populations [18]. The synergistic effect observed in our analysis—reflected by an odds ratio of 19.3 for the interaction between high alcohol consumption and severe depression—mirrors multiple biological and behavioral mechanisms that have been extensively documented in the literature [30,31,32].

### 4.2. Pathophysiological Mechanisms of the Alcohol–Depression–Diabetes Interaction

The bidirectional relationship between depression and diabetes has been well documented in the recent literature, with studies demonstrating that individuals with depression have a 60% higher risk of developing T2DM, while those with diabetes have a twofold risk of developing depression [31,33]. This bidirectional relationship is mediated by common pathophysiological mechanisms, including chronic inflammation, oxidative stress, and HPA dysfunction [33,34].

Regarding alcohol consumption, studies demonstrate that it can induce insulin resistance through multiple pathways [35,36,37]. Volkow et al. have shown that alcohol consumption causes a 20% decrease in cerebral glucose metabolism in heavy drinkers compared with 9% in controls, suggesting a fundamental alteration in glucose utilization as an energy substrate [38]. This alteration is associated with the use of acetate as an alternative energy substrate, a mechanism that may contribute to the disruption of glucose homeostasis.

Beyond the pathophysiological mechanisms, it is essential to consider the behavioral link that connects the psychological state to the metabolic state. Alcohol consumption and depression are known for their ability to disrupt healthy eating behaviors, leading to unbalanced eating. A central mechanism in this process is the alteration of intuitive nutrition—the individual’s ability to recognize and respond to internal signals of hunger and satiety. A recent study by Curis et al. 2023 [39] highlights the role of intuitive nutrition as an essential factor in maintaining emotional and nutritional balance. Therefore, it can be postulated that the synergistic effect of alcohol and depression is mediated, at least in part, by the erosion of this capacity for dietary self-regulation, which leads to chaotic nutritional choices and hyperglycemia [39].

To explore potential explanatory mechanisms, we conducted a mediation analysis to assess whether depression severity mediates the relationship between alcohol consumption and glycemia. Although alcohol use was positively associated with higher depression scores, and depression was linked to increased fasting glucose, the indirect effect (a × b) was not statistically significant. This suggests that depression does not significantly mediate the effect of alcohol consumption on hyperglycemia in this clinical sample.

These findings partially contrast with previous studies that proposed depression as a potential mediator in the relationship between substance use and metabolic dysregulation [40,41], where inflammatory and behavioral mechanisms were hypothesized to explain this link. However, other studies have also reported inconsistent evidence regarding the mediating role of depression, especially in psychiatric populations where symptom overlap and medication status may confound these pathways [42].

One possible explanation is that alcohol and depression exert independent and additive effects on glucose metabolism through distinct physiological pathways—such as hepatic insulin resistance in the case of alcohol and HPA axis dysregulation in the case of depression. Alternatively, unmeasured variables—such as stress biomarkers, cortisol levels, or nutritional deficits—may play a stronger mediating role than depression itself. Further longitudinal studies including biological and behavioral mediators are needed to clarify these complex interactions.

### 4.3. Dysfunction of HPA Axis

One of the central mechanisms explaining the interaction observed in our study is the dysfunction of the HPA axis [34]. Both excessive alcohol consumption and depression activate this axis, leading to increased cortisol levels [34,43]. Cortisol promotes hepatic gluconeogenesis and induces insulin resistance, mechanisms that may explain the elevated blood glucose levels observed in patients with both risk factors. Studies demonstrate that depression is associated with a flattened diurnal cortisol curve, which predicts the incidence of diabetes and higher levels of HbA1c [34]. This dysfunction of the HPA axis may be exacerbated by alcohol consumption, which acutely activates the sympathetic system and can lead to chronic autonomic dysfunction [37].

### 4.4. Systemic Inflammation and Neuroinflammation

Chronic inflammation represents another crucial mechanism in explaining the observed interaction. Excessive alcohol consumption induces both a systemic and central nervous system inflammatory response, with increased levels of pro-inflammatory cytokines such as TNF-α, IL-6, and IL-1β [44,45,46]. These cytokines can cross the blood–brain barrier and affect neuronal function, contributing to the development of depression [47].

Leclercq and colleagues demonstrated that altered gut microbiota in patients with alcohol use disorder induces metabolic changes that affect adipose tissue and the liver, resulting in the decreased synthesis of β-hydroxybutyrate and neurobiological and behavioral alterations. These changes may contribute to the development of both depression and metabolic disturbances [48].

A particularly relevant aspect for our study is the demonstration that patients with alcohol dependence and depressive symptoms exhibit specific cytokine responses to stress. The study by Fox and colleagues showed that the stress-induced suppression of TNF-α and TNFR1 predicts the severity of alcohol consumption after treatment only in patients with alcohol dependence and subclinical depression [43].

### 4.5. Neurotransmitter Systems

Alterations in neurotransmitter systems represent an important mechanism through which alcohol consumption and depression may interact to affect glucose metabolism [49,50,51]. The serotonergic system plays a key role in modulating cerebral glucose levels, and polymorphisms in the serotonin transporter gene (5-HTT) have been associated with depression in individuals with diabetes [51].

Alcohol impacts multiple neurotransmitter systems, including serotonin, dopamine, GABA, and glutamate [50,52,53]. These alterations may contribute to the development of depression and may indirectly affect glucose metabolism by influencing eating behaviors and treatment adherence [52].

The GABAergic system is particularly relevant as alcohol initially enhances the activity of GABA-A receptors, but chronic consumption leads to the downregulation of these receptors and withdrawal-induced hyperexcitability [53,54]. This GABAergic dysfunction may contribute to both depressive symptoms and metabolic disturbances.

### 4.6. Therapeutic Implications and Integrated Management

The clinical implications of our findings are significant and underscore the need for integrated management strategies for patients at risk. While our study focuses on identifying synergistic risk, further research is essential to establish the most effective treatment approaches. For example, recent studies precisely explore these aspects, analyzing the response to short- and long-term psychological interventions and highlighting the complexity of shaping therapeutic response in patients with alcohol–depression comorbidity. Such studies are important to translate etiological findings, such as those presented here, into effective clinical protocols [55].

In addition to the pathophysiological mechanisms discussed, it is important to consider the role of psychological resources that may act as protective factors or, in their absence, may worsen patients’ vulnerability. While our study focused on risk factors such as depression, the literature emphasizes the importance of protective factors such as optimism. It is considered a fundamental resource and a form of the manifestation of positive affectivity, the presence or absence of which is influenced by the life context and situational stressors, such as pandemics [56]. Thus, the devastating synergistic effect of alcohol and depression could be explained not only by their direct impact but also by the erosion of such protective psychological resources. In addition to strictly biological mechanisms, it is crucial to analyze psychological constructs that may mediate the relationship between depression and risk behaviors, such as excessive alcohol consumption. A complementary perspective is offered by studies in the field of developmental psychology, which demonstrate the fundamental role of self-esteem as a mediating factor between the external environment (e.g., parenting styles) and adaptive or maladaptive outcomes.

### 4.7. Limitations

This study has several limitations that should be acknowledged. First, its cross-sectional design precludes any inference of causality between alcohol consumption, depression, and glycemic status. Longitudinal studies are needed to establish temporal sequences and potential causal pathways.

Second, the assessment of alcohol use was based on the total AUDIT score, which, although validated and widely used, does not capture specific drinking patterns such as binge or heavy episodic drinking. These patterns may have distinct metabolic consequences, as suggested by previous studies, and should be explicitly measured in future research.

Third, while we excluded patients with a prior diagnosis of diabetes or alcohol dependence, we cannot fully exclude the influence of undiagnosed prediabetes or subclinical liver dysfunction on the observed associations. Moreover, fasting glucose was measured only once, without confirmation through repeated tests or HbA1c, which limits the diagnostic reliability of hyperglycemia status.

Fourth, although patients were clinically stable at the time of data collection, we cannot entirely rule out the influence of hospitalization-related stress on glucose levels, especially in individuals with elevated psychiatric symptomatology. While we attempted to minimize this by excluding patients in an acute crisis or severe psychotic agitation, residual stress effects may still be present.

Finally, the study was conducted in a single psychiatric hospital in Romania, which may limit the generalizability of the findings to other populations or health systems.

Despite these limitations, this study provides novel evidence regarding the interaction between alcohol use and depression as risk factors for hyperglycemia, with implications for integrated screening in psychiatric settings.

## 5. Conclusions

The results of our study are consistent with a substantial body of the scientific literature demonstrating complex interactions between alcohol consumption, depression, and glucose metabolism. The multiple pathophysiological mechanisms identified in the literature—HPA axis dysfunction, chronic inflammation, neurotransmitter alterations, oxidative stress, and epigenetic changes—provide a solid theoretical foundation for the synergistic effect observed in our study.

The dramatic effect of the interaction between high alcohol consumption and severe depression on the risk of hyperglycemia—with an odds ratio of 20 and a prevalence exceeding 95% in the group with both risk factors—underscores the critical importance of an integrated approach to the management of these patients. The scientific literature supports this conclusion and highlights the necessity of systematic screening, integrated interventions, and ongoing research to optimize prevention and treatment strategies for this vulnerable population.

## Figures and Tables

**Table 1:** Baseline characteristics of the study population.

Variable	Total (n = 172)	Men (n = 113)	Women (n = 59)	*p*-Value
Age (years)	43.5 ± 12.3	44.1 ± 11.7	42.3 ± 13.4	0.412
BMI (kg/m^2^)	27.8 ± 4.2	27.5 ± 4.5	28.4 ± 3.7	0.264
Glycemia (mg/dL)	144.7 ± 26.2	146.2 ± 27.1	142.0 ± 24.5	0.305
Hyperglycemia prevalence	58.7%	61.9%	52.5%	0.305
GGT (U/L)	59.7 ± 17.6	61.2 ± 18.3	56.8 ± 16.0	0.172
Severe depression (BDI > 25)	45.9%	39.8%	52.5%	0.152
High alcohol use (AUDIT > 20)	54.1%	59.3%	45.8%	0.126

**Table 2:** Logistic regression models for predicting hyperglycemia risk (adjusted for age, sex, and GGT).

Model	Comparison	OR (95% CI)	*p*-Value
1	High Alcohol vs. Low	1.38 (0.61–3.09)	0.441
2	Severe Depression vs. None	1.30 (0.51–3.31)	0.582
3	Interaction (High Alcohol × Severe Depression)	19.30 (3.22–115.81)	0.001
4	Both Risk Factors vs. Neither	28.85 (4.81–173.02)	<0.001

**Table 3:** Mean blood glucose levels across combinations of alcohol consumption and depression severity.

High Alcohol	High Depression	Mean	Std	Count
0	0	133.9	25.9	50
0	1	134.5	24.1	28
1	0	143.3	25.5	46
1	1	163.3	17.4	48

## Data Availability

The original contributions presented in this study are included in the article. Further inquiries can be directed to the corresponding author(s).

## Abbreviations

The following abbreviations are used in this manuscript:

T2DM	Type 2 Diabetes Mellitus
GGT	gamma-Glutamyl Transferase
BDI	Beck Depression Inventory
AUDIT	Alcohol Use Disorder Identification Test
HPA	Hypothalamic–Pituitary–Adrenal
5-HTT	Serotonin Transporter Gene
BMI	Body Mass Index
